# Fractional laser ablation for the targeted cutaneous delivery of an anti-CD29 monoclonal antibody – OS2966

**DOI:** 10.1038/s41598-018-36966-0

**Published:** 2019-01-31

**Authors:** Maria Lapteva, Sergio del Río-Sancho, Eric Wu, W. Shawn Carbonell, Christof Böhler, Yogeshvar N. Kalia

**Affiliations:** 1 School of Pharmaceutical Sciences, University of Geneva & University of Lausanne, Geneva, Switzerland; 2 OncoSynergy, Inc., Greenwich, CT, USA; 3 Pantec Biosolutions, Ruggell, Liechtenstein

## Abstract

Monoclonal antibodies targeting cytokines are administered parenterally for the systemic treatment of severe psoriasis. However, systemic exposure to the biologic increases the risk of side-effects including immunosuppression, whereas only a small fraction of the active molecules actually reaches the target organ, the skin. This preclinical study examines the feasibility of delivering a humanized anti-CD29 monoclonal antibody (OS2966) topically to skin using minimally-invasive fractional laser ablation. This approach would enable the targeted use of a biologic for the treatment of recalcitrant psoriatic plaques in patients with less widespread disease while minimizing the risk of systemic exposure. First, the effect of a wide range of laser poration conditions on skin permeation and deposition of OS2966 was tested *in vitro* to determine optimal microporation parameters. Subsequently, confocal laser scanning microscopy was employed to visualize the distribution of fluorescently-labelled OS2966 in skin. The results demonstrated that delivery of OS2966 into and across skin was feasible. Above fluences of 35.1 J/cm^2^, skin deposition and permeation were statistically superior to passive delivery reaching values up to 3.7 ± 1.2 µg/cm^2^ at the most aggressive condition. Selective targeting of the skin was also possible since ≥70% of the OS2966 was delivered locally to the skin. Although nanogramme quantities were able to permeate across skin, these amounts were orders of magnitude lower than levels seen following subcutaneous or intravenous injection and would result in minimal systemic exposure *in vivo*. The diffusion of fluorescently-labelled OS2966 into the skin surrounding the pores was clearly higher than in intact skin and demonstrated the feasibility of delivering the antibody at least as deep as the dermo-epithelial junction, a critical border region where inflammatory cells cross to promote disease progression. These preliminary results confirm that fractional laser ablation can be used for the cutaneous delivery of OS2966 and now preclinical/clinical studies are required to demonstrate therapeutic efficacy.

## Introduction

Plaque psoriasis is an autoimmune skin condition, affecting approximately 25 million people in North America and Europe^1^. The physiopathology of the disease is complex and balanced by both environmental and genetic factors^2^. The skin of a psoriatic patient is deregulated in terms of both innate and acquired immunity. Cytokines including a wide range of interleukins, interferon-γ and TNF-α (tumour necrosis factor alpha) stimulate the proliferation of keratinocytes which in turn produce more antimicrobial peptides (β-defensins and psoriasin) and chemokines attracting neutrophils and T-cells into the viable epidermis. Feedback loops are responsible for further keratinocyte, fibroblast and endothelial cell activation leading to increased collagen synthesis and angiogenesis^2^.

Several therapies are available for the treatment of the disease^3^. Topical application of corticosteroids, vitamin D derivatives or their combination can lead to plaque clearance^4^ in mild and moderate disease states. Phototherapies using narrowband and broadband UV-B radiation alone or UV-A radiation combined with psoralen photosensitization are effective in moderate to severe plaque type psoriasis^5^. In the case of severe psoriasis, treatments involving oral administration of immunosuppressants such as ciclosporin A or tacrolimus are needed^4^. A range of biopharmaceuticals such as monoclonal antibodies (mAbs) or fusion proteins targeting the chemokines implicated in psoriasis pathogenesis have reached the market^6^. TNF-α was among the first cytokines to be targeted using infliximab, adalimumab and etanercept, followed by the anti-interleukin12–23 ustekinumab (mAb targeting the common p40 chain of these cytokines)^2^. More recently, secukinumab an anti-interleukin17A (IL17A) antibody was approved by the Food and Drug Administration (FDA)^7^. However, due to their proteic nature and associated physicochemical properties, these drugs are administered either by intravenous or subcutaneous injection and are only indicated for patients that are candidates for systemic therapy.

An optimal therapeutic agent for psoriasis should be (i) effective in plaque clearing and improving PASI scores (PASI: Psoriasis Area and Severity Index, a diagnostic tool used for the measurement of severity of psoriasis), (ii) preferably administered by the topical route to minimize systemic side-effects and (iii) interact selectively with molecular targets implicated in the deregulated skin immune response. Topically applied corticosteroids meet the first and second criteria; however, the lack of selectivity leads to numerous side effects such as cutaneous atrophy, formation of telangiectasia, steroid rosacea, perioral dermatitis and increased risk of skin infections^8^. Similarly, psoralen and ultraviolet A (PUVA) therapy may lead to increased risk of skin cancer^9^. Orally administered agents meet only the first condition and can provoke a series of severe side effects due to systemic exposure: renal and cardiac toxicity can be mentioned as examples for ciclosporin A. Biopharmaceutical therapies are promising because of their selective binding to receptors implicated in the immune response; however, their invasive parenteral administration and therefore distribution throughout the whole organism may lead to serious side effects such as anaphylaxis^10^ or even unpredictable life-threatening “cytokine storms”^11^. They are currently only indicated for severe disease states. In order to meet the three conditions of an ideal psoriasis therapy, and enable their more widespread use, selective biopharmaceuticals such as mAbs should be administered topically to treat more localized and less widespread disease conditions – targeted cutaneous delivery would increase efficacy, expand their potential use and avoid systemic exposure.

Many efforts have been made to satisfy the requirements of such an ideal psoriasis therapy. For instance a recent Phase II clinical investigation by Tsianakas *et al*.^12^. tested the topical and intradermal administration of DLX105 (Cell Medica Ltd; developed by Delenex Therapeutics AG), a novel 26 kDa anti-TNF-α humanized single-chain (scFv; single-chain variable fragment) monoclonal antibody, to patients with chronic plaque-type psoriasis. Intradermal injections of DLX105 into the psoriatic lesions led to a significant decrease in mean local PASI scores over baseline after treatment for 14 days, together with decreased K16, Ki67 biomarkers, epidermal thickness and mRNA levels of several proinflammatory cytokines. However, topical administration of 0.5% DLX 105 formulated in a hydrogel did not yield comparable results. Indeed, only the reduction of proinflammatory cytokine mRNA was observed without any response at the histological and clinical scales. Tape-stripping of the lesion did not bring any improvement. The authors suggested that DLX 105 could penetrate into the epidermis via the impaired barrier of the lesion, but could not reach the dermis in sufficient amounts to provoke a clinically meaningful result – thus, leading to the conclusion that: “increasing drug concentration in the dermis by biological, chemical or physical methods and, maybe, the development of a more potent follow-on TNF-α blocker might result in improved clinical efficacy”^12^.

It is well known that only small molecules with the appropriate physicochemical properties can diffuse passively across the *stratum corneum*, the uppermost skin layer which is the principal barrier to transport^13^: a physical or chemical enhancement technique is needed to transiently breach the *stratum corneum* barrier to enable delivery of drugs with “less ideal” properties. It has been shown that minimally-invasive erbium-doped yttrium aluminium garnet (Erbium:YAG) fractional laser ablation can be used to deliver functional proteins to skin, e.g. cytochrome C (12.4 kDa)^14^, recombinant human growth hormone (hGH; 22 kDa)^14,15^, urinary follicle stimulating hormone (FSH; 30 kDa)^14^, FITC-labelled bovine serum albumin (FITC-BSA; 70 kDa)^14^ and more interestingly anti-thymocyte globulin and basiliximab (155 kDa)^16^. Furthermore, it was also able to deliver macromolecular antigens such as Recombinant Phl p 5, a grass pollen allergen (38 kDa),ovalbumin (44 kDa), or betagalactosidase into the skin for transcutaneous immunization *in vivo*^17^.

**Figure 1:**
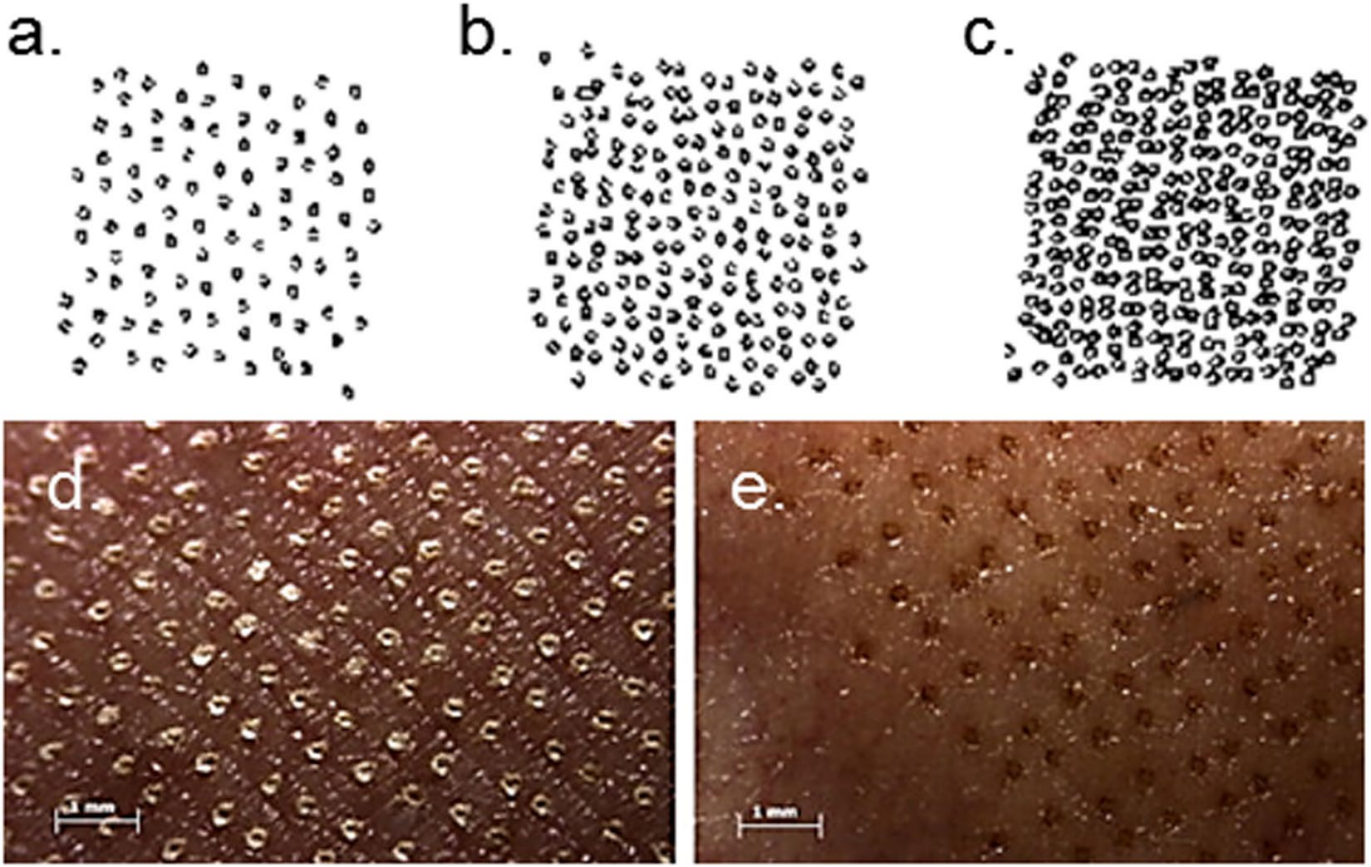
Typical laser poration pattern (1 cm^2^) for different fractional ablated areas: (**a**) 5%, (**b**) 10%, (**c**) 15%, and magnification (20x) of porated skin at (**d**) a fluence of 23 J/cm^2^ (pulse length 75 μs, repetition rate 400 Hz, stack of 5 pulses per pore) and (**e**) at 39 J/cm^2^ (pulse length 125 μs, repetition rate 300 Hz, stack of 5 pulses per pore). Reproduced with permission from Gratieri *et al*.^13^.

Lasers used for drug delivery are derived from devices used for skin resurfacing applications for the local treatment of medical conditions (e.g., keloids or warts) or aesthetic dermatology (e.g., wrinkle reduction or removal of age spots)^18^. Low intensity Erbium:YAG (solid state erbium-doped yttrium aluminium garnet) lasers emitting light at 2940 nm are routinely used for skin ablation^18^. This wavelength corresponds to an absorption band of water in the infrared spectrum. As the laser pulse imparts energy to the skin, water molecules vibrate and start heating-up. Depending on the temperature reached, four effects may occur: heating (37–60 °C), coagulation (60–65 °C), drying (90–100 °C), and vaporization (>100 °C)^18^. Each pulse is able to ablate a reproducible amount of tissue and thus the pore depth can be controlled^19^. Using this technology, Pantec Biosolutions AG (Ruggell, Liechtenstein) developed the P.L.E.A.S.E.^®^ system (Precise Laser Epidermal System) for delivery of low and high molecular weight molecules. The device is able to create an array of 150 µm diameter micropores on a small skin area. The pore depth is monitored by modulating i) the number of pulses per pore and ii) pulse energy or fluence (J/cm^2^). The latter depends on the pulse duration (µs) and repetition rate (Hz). To obtain minimally-invasive painless ablation, the pore depth must be limited so as not to reach the sensitive nerve endings situated in the dermis. Finally, the number of pores created per unit area determines the fraction of skin that is ablated and this is defined as the fractional ablated area (%). Fig. 1a–c show the different pore distribution patterns obtained when using different fractional ablated areas and Fig. 1d,e show the skin aspect after fractional laser ablation with the pulsed P.L.E.A.S.E.^®^ Professional device: Fig. 1d shows an example of coagulated tissue and Fig. 1e shows pores where the borders have carbonized tissue (black perimeters) as the applied fluence increases. Moreover, the tolerability of the P.L.E.A.S.E.^®^ treatment has been demonstrated *in vivo*^20,21^.

OS2966 is a neutralizing anti-CD29 (Cluster of differentiation 29 or β1-integrin subunit) mAb, developed for the treatment of glioblastoma by OncoSynergy, Inc. Integrins are proteins responsible for cell-matrix and cell-cell interactions. They assemble into α-β dimers. β_1_ integrins constitute the majority of integrins and bind to extra-cellular matrices (ECM), thus in healthy skin they are mostly expressed in the basal layer of keratinocytes where they are known to be implicated in cell adhesion to the basal lamina. The abnormal expression of integrins in upper epidermal layers is linked to psoriasis-like phenotype formation^22^.

Alongside this aspect of psoriasis pathogenesis, and as already mentioned above, unlike in healthy skin, T cells can be found in the epidermis of psoriatic patients^1^, meaning that they need to cross the dermo-epithelial junction. Conrad *et al*. showed that epidermal but not dermal T-cells in psoriatic lesions express α_1_β_1_ integrin known to bind to collagen IV (in CD3^+^ T-cells)^23^. The xenotransplantation mouse model showed that the increase in the α_1_β_1_^+^CD3^+^ T-cells numbers in the epidermis of prepsoriatic skin led to the onset of a psoriatic phenotype. Most interestingly, when the α_1_ subunit was blocked by an anti-α_1_ mAb *in vitro*, the ability of α_1_β_1_^+^ T-cells to cross collagen IV was significantly inhibited. Those results were confirmed *in vivo* in the xenotransplantation mouse model: the xenografts injected (sub. cut.) with the anti-α_1_ mAb were characterized by a significant decrease in acanthosis and paillomathosis^23^.

Although α_1_β_1_ inhibition alone was efficacious in the above studies, the complexity of the psoriatic disease process will likely mean that modulation of more than one integrin heterodimer is required in the clinic. Indeed, there are twelve known CD29 integrin heterodimers mediating adhesion to myriad ECM including multiple collagen receptors (e.g., α_1_β_1_, α_2_β_1_, α_8_β_1,_ α_10_β_1_) and fibronectin receptors (e.g., α_5_β_1_, α_8_β_1_, α_v_β_1_). All are implicated in dynamic tissue remodelling including the inflammation, fibrosis, and angiogenesis seen in psoriasis^24^. OS2966 is the first pan-CD29 inhibiting therapeutic candidate in development and is thus functionally equivalent to twelve separate antibodies for more effective modulation of the inflammatory process.

Taking this data into consideration the local application of OS2966 and its binding to CD29 could be of therapeutic interest in the treatment of psoriasis and inhibition of T-cell migration to the epidermis. Consequently, the objective of this preclinical study was to investigate the effect of P.L.E.A.S.E.^®^ laser microporation conditions on the delivery of OS2966, a humanized IgG1 (immunoglobulin G1) monoclonal antibody, into and across skin and to visualize its biodistribution within the membrane. The *in vitro* evaluation of delivery was used to identify the optimal conditions for subsequent clinical studies and was also intended to help to determine the number and proximity of microporation sites necessary to enable delivery of therapeutic amounts of the drug candidate.

## Results

### Cutaneous delivery experiments

#### Effect of laser poration parameters on OS2966 delivery at fixed donor concentration and fractional ablated area

**Figure 2:**
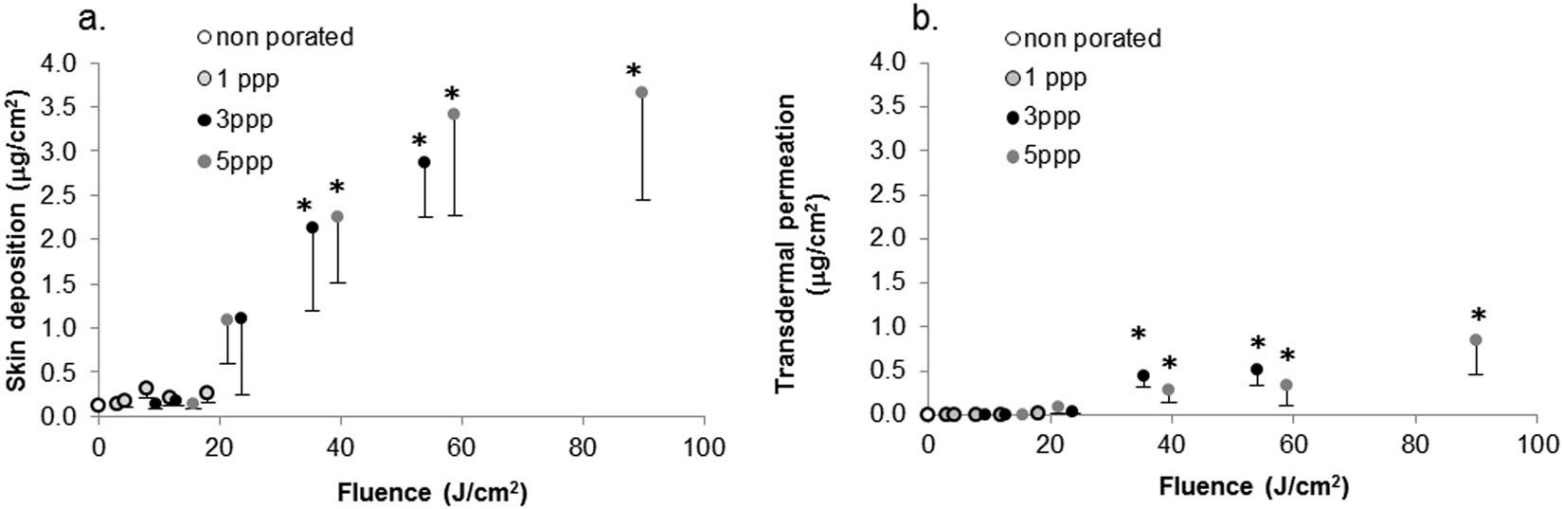
Effect of laser fluence on (**a**) skin deposition and (**b**) transdermal permeation of OS2966 after formulation application on porated skin for 12 h (mean ± SD; *p < 0.05 when compared to non-porated; ppp: pulse per pore).

Topical deposition in skin and transdermal permeation of OS2966 as a function of laser fluence (J/cm^2^) are presented in Fig. 2.

The results suggested that a fluence of 20 J/cm^2^ represented a threshold to OS2966 delivery. When skin was porated at a fluence below 20 J/cm^2^, antibody deposition in the skin was not statistically different from the non-porated control conditions (Fig. 2a). However, for fluences of 20 J/cm^2^ and above, skin deposition increased almost linearly starting at a fluence of 21.3 J/cm^2^ with a deposition of 1.094 ± 0.491 µg/cm^2^ to reach 3.665 ± 1.224 µg/cm^2^ at the most aggressive conditions. Both skin deposition and transdermal permeation of the antibody became significantly higher than the control at a fluence of 35.3 J/cm^2^. At fluences ≥35.3 J/cm^2^, OS2966 permeated across skin (0.445 ± 0.131 μg/cm^2^) and its permeation increased to 0.851 ± 0.397 μg/cm^2^ at the most aggressive condition.

#### Effect of fractional ablated area on OS2966 delivery using fixed number of pulses per pore and donor concentration

In order to study the influence of fractional ablated area (FAA) on OS2966 delivery, the skin was porated to remove increasing amounts of skin per unit area (5% = 100 pores; 10% = 200 pores; 15% ≈ 300 pores per cm^2^)(Fig. 1), at a fixed donor concentration of 1 mg/ml and number of pulses per pore (3ppp).

**Figure 3:**
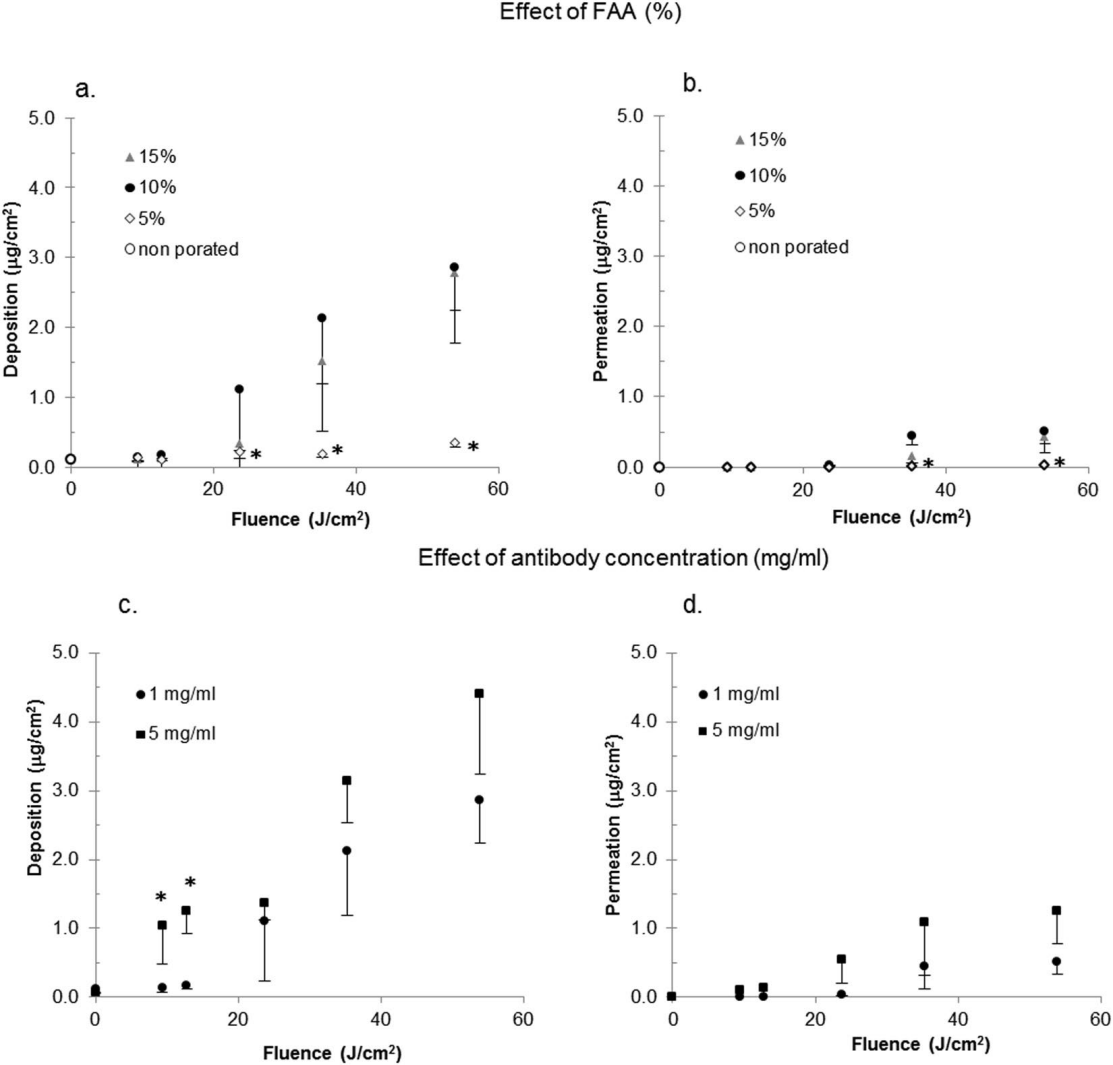
Effect of fractional ablated area (FAA; % of skin surface ablated by laser per cm^2^) on (**a**) skin deposition and (**b**) skin permeation of OS2966 and effect of increased antibody content on (**c**) skin deposition and (**d**) skin permeation of OS2966. (data expressed as mean ± SD; *p < 0.05 when compared to the initial condition: black circle; 1 mg/ml,10% FAA, 3ppp; ppp: pulse per pore).

Figure 3a,b show the influence of FAA on OS2966 deposition and permeation as a function of applied fluence. Consistent with previous results, skin deposition was low for fluences below 20 J/cm^2^ and was not influenced by the FAA. At fluences above 20 J/cm^2^ the increase of FAA from 5 to 10% resulted in a significant increase in skin deposition (i) from 0.217 ± 0.091 µg/cm^2^ to 1.103 ± 0.491 µg/cm^2^, (ii) from 0.188 ± 0.056 µg/cm^2^ to 2.131 ± 0.940 µg/cm^2^, and (iii) from 0.351 ± 0.066 µg/cm^2^ to 2.863 ± 0.618 µg/cm^2^ for fluences of 23.7; 35.3 and 53.9 J/cm^2^, respectively. The further increase of FAA to 15% did not yield an increase in OS2966 delivery. Permeation of OS2966 was observed at 23.7 J/cm^2^ with FAA of 10% and 15% and ranged from 0.032 ± 0.010 µg/cm^2^ to 0.513 ± 0.183 µg/cm^2^.

#### Effect of donor concentration on OS2966 delivery using fixed number of pulses per pore and FAA

Figure 3c,d compare skin deposition and skin permeation of OS2966 using two different donor concentrations: 1 and 5 mg/ml at 3 ppp and 10% FAA. Increasing the OS2966 concentration from 1 to 5 mg/ml in the formulation resulted in significantly increased skin deposition for groups treated with 9.4 and 12.8 J/cm^2^ (from 0.138 ± 0.061 µg/cm^2^ to 1.036 ± 0.547 µg/cm^2^ and from 0.174 ± 0.055 µg/cm^2^ to 1.249 ± 0.325 µg/cm^2^ respectively). In contrast, the application of a 5 mg/ml formulation did not significantly increase skin deposition porated with 23.7; 35.3 and 53.9 J/cm^2^. It should be noted that these treatments led to an increased permeation, ranging from 0.544 ± 0.336 µg/cm^2^ to 1.246 ± 0.464 µg/cm^2^.

### Skin distribution of labelled OS2966

**Figure 4:**
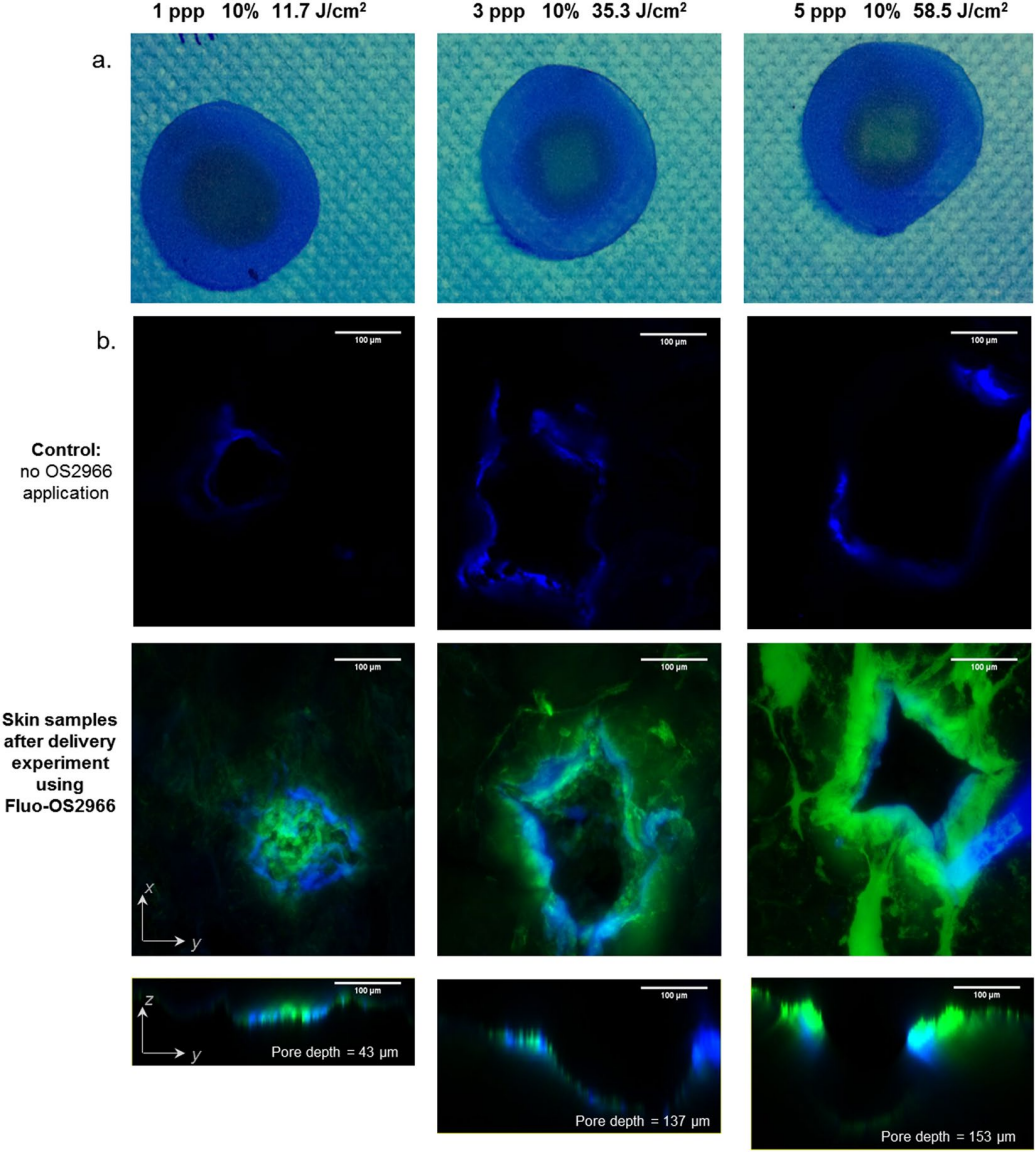
(**a**) UV (366 nm) illuminated skin samples following permeation experiment, (**b**) Alexa 488-OS2966 (green) and Hoechst 33258 / auto-fluorescence of skin (blue) signals in healthy porated porcine skin following permeation experiment. Scale bar = 100 μm. ppp: pulse per pore.

The effect of laser poration could be observed at a macroscopic scale (Fig. 4a). The region of skin treated with 35.3 J/cm^2^ and 58.5 J/cm^2^ (at 175µs/200 Hz) appeared increasingly brighter than the adjacent intact skin when placed under a UV lamp (366 nm) (Fig. 4a). The confocal laser scanning microscopy (CLSM) study enabled the visualization of pores created with the different laser treatments (Fig. 4b). Hoechst 33258 (blue) enabled visualization of the skin cells, whereas the green signal came from the Alexa 488-OS2966. The pore depths were 43 µm, 137 µm and 153 µm for 1 ppp, 3 ppp and 5 ppp, respectively (at 175µs/200 Hz). Consequently, the use of 5 ppp, 10%; 58.5 J/cm^2^, which was the second most aggressive condition enabled the creation of micropores that were most likely reaching the upper dermis (dermis being considered to be situated 150–160 µm below skin surface^25,26^).

**Figure 5:**
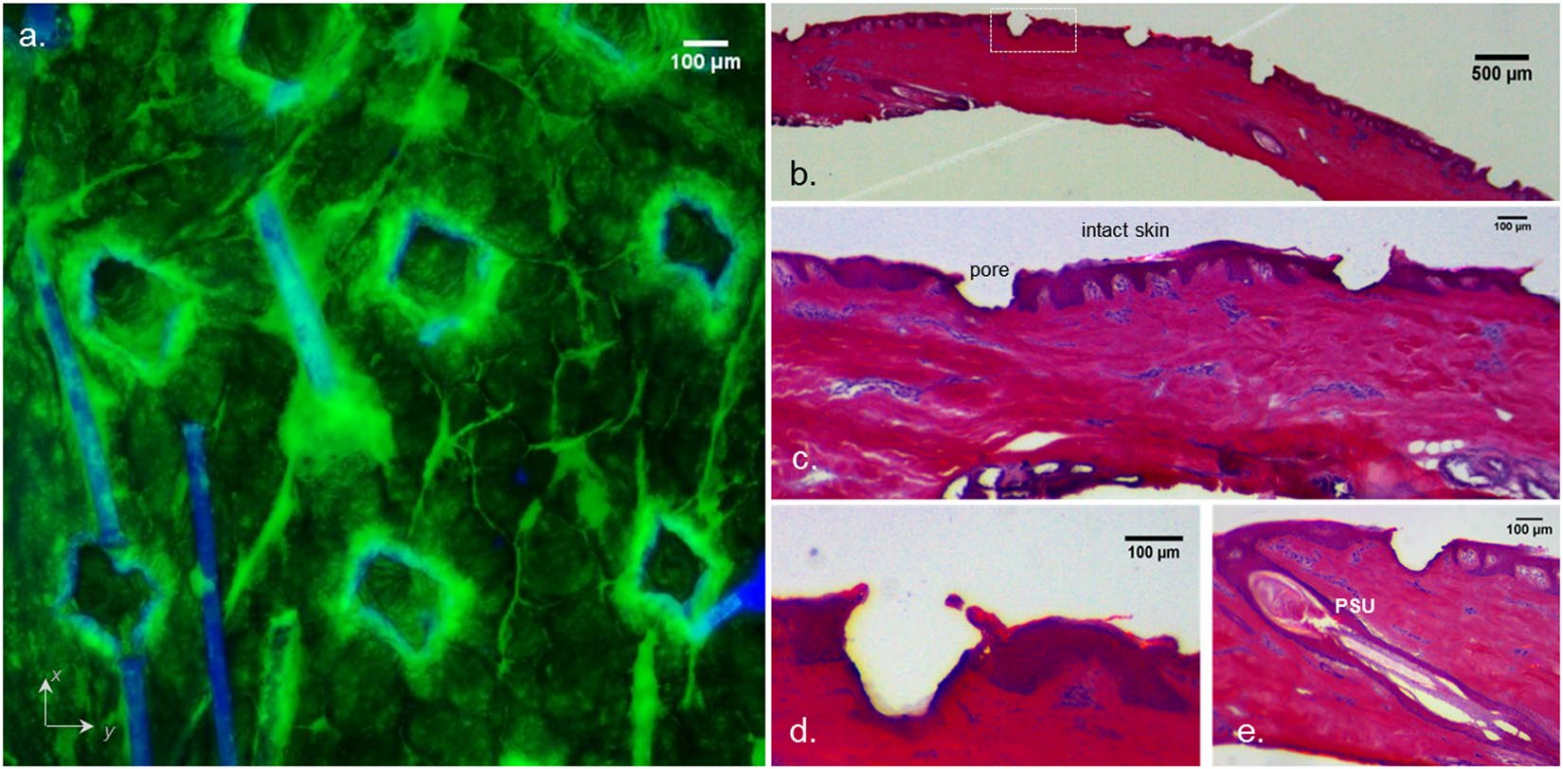
(**a**) CLSM Wide field (x5 objective) view on healthy porcine skin porated with 5 ppp, 10%; 58.5 J/cm^2^. ppp: pulse per pore Green signal: Alexa 488-OS2966; blue signal: Hoechst 33258 / skin auto-fluorescence. Scale bar = 100 μm. (**b**–**e**) optical microscopy Hematoxylin and eosin stain of porcine skin porated with 5 ppp, 10%; 58.5 J/cm^2^, (**b**) and (**c**) wide field view of porated skin (6.3x and 20x), (**d**) magnification of one pore from panel (**b**) (White square) (40x), (**e**) laser created pore adjacent to a pilosebaceous unit (PSU) (40x). Scale bar = 100 or 500 μm

This was confirmed by the histological observation of porated skin samples (Fig. 5b–e; 5 ppp, 10%; 58.5 J/cm^2^) The average pore depth was found to be 154.6 ± 35 µm; n = 9 and was consistent with the CLSM observations.

Figure 5a shows a broad view of the porated skin surface (xy-plane) after application of the Alexa 488-OS2966 formulation. It appears that the green signal originating from the labelled antibody was mainly localized around and inside the pores as well as in the inter-cluster regions^27^. CLSM microscopy also enabled three dimensional reconstructions to be made by combining images of each confocal plane.

**Figure 6:**
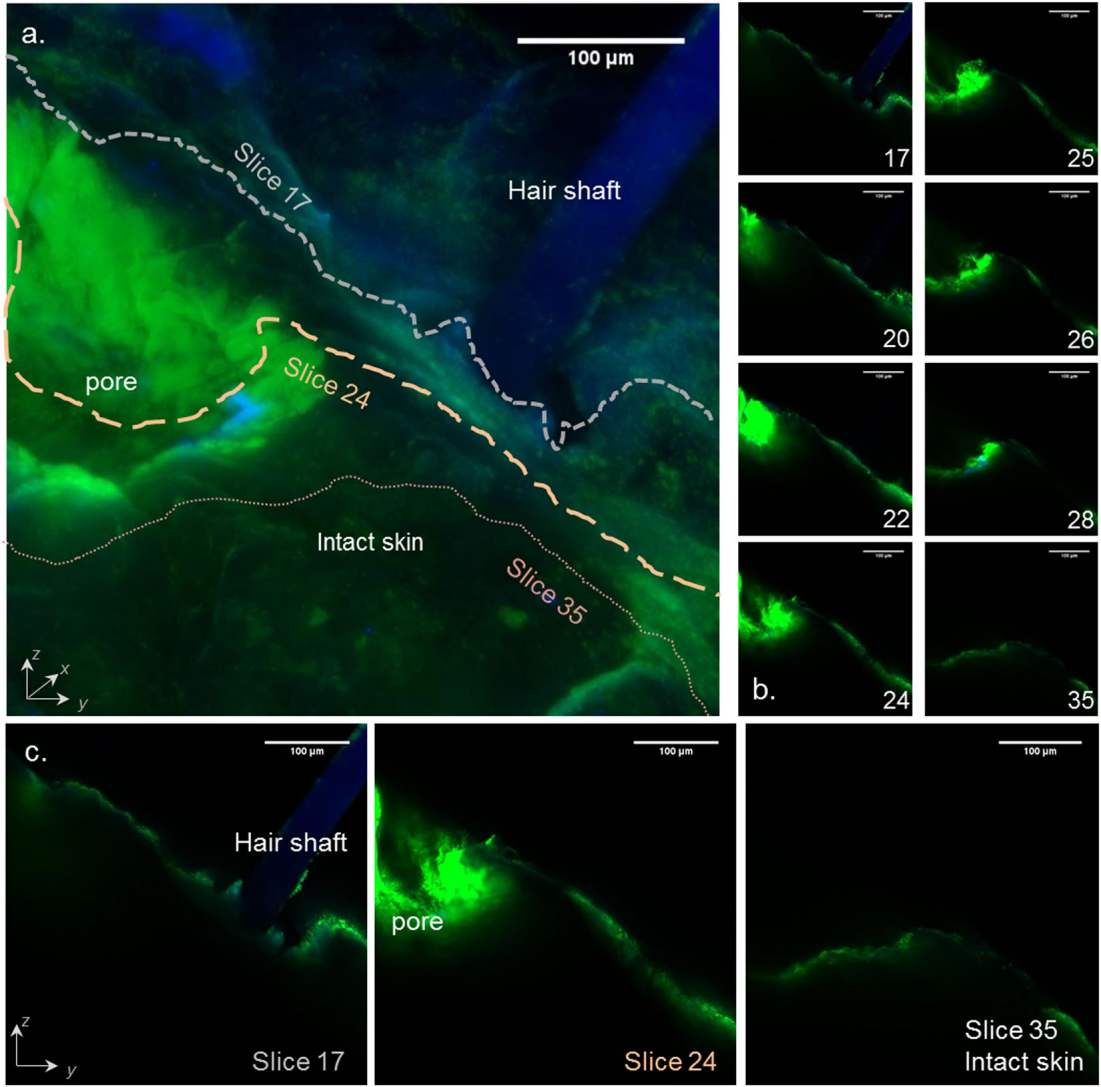
(**a**) Three-dimensional reconstruction of healthy porated porcine skin surface (treated with 5 ppp, 10%; 58.5 J/cm^2^), (**b**) and (**c**) selected slices showing cross-sections of skin with a hair shaft, a pore and an intact area of skin. Green signal: Alexa 488-OS2966; blue signal: Hoechst 33258 / skin auto-fluorescence. Scale bar = 100 μm. ppp: pulse per pore.

Thus, Fig. 6a shows the three dimensional reconstruction of skin presenting a laser-created pore and a hair follicle. Each component “slice” making up the image can be looked at individually and thus Fig. 6b,c indicate that (i) the Alexa 488-OS2966 did not enter intact skin (slice 35), (ii) the diffusion of Alexa 488-OS2966 into the skin in the surroundings of the pores and beneath was clearly higher than in intact skin (slice 24), and (iii) hair follicles did not seem to be a preferential pathway (slice 17). Overall, the results suggested that the laser-created pores were the only penetration pathway for OS2966 into the skin.

## Discussion

The variation of laser settings such as pulse duration, repetition frequency and number of pulses used to create the pores enabled the controlled cutaneous delivery of a 150 kDa monoclonal IgG1 therapeutic antibody. The results suggested that poration with fluences below 20 J/cm^2^ did not enhance skin deposition or permeation of OS2966 in comparison to control. The CLSM images showed that when mild poration conditions were used (1 ppp, 11.7 J/cm^2^; Fig. 4b), the ablation depth was about 40 μm, which corresponded to the superior layers of the viable epidermis. In contrast, when skin was porated at fluences above 20 J/cm^2^, OS2966 deposition and permeation increased linearly. CLSM microscopy confirmed the presence of pores deeper than 120 μm (i.e. lower layers of viable epidermis and perhaps entering the upper dermis) in which the labelled OS2966 was able to accumulate (Figs 4b and 6c) and then diffuse to the deeper dermis. These observations suggest that *stratum corneum* is not the only barrier to the delivery of the OS2966 and this barrier to diffusion is situated in the viable epidermis. It is probable that for a molecule as large as OS2966 the *stratum granulosum* and *spinosum* with its desmosomes can also constitute a diffusional barrier^28^.

There are few reports in the literature that describe topical delivery of antibodies and quantify the amount present in the skin. The results described here are comparable to those obtained previously with anti-thymocyte globulin (ATG; a polyclonal antibody) and basiliximab using a prototype of the P.L.E.A.S.E.^®^ laser^16^. Indeed Yu *et al*. obtained a total delivery of 1.18 ± 0.10 µg/cm^2^ and 1.92 ± 0.48 µg/cm^2^ for ATG and basiliximab respectively (Conditions: 300 pores over diffusion area, 45.3 J/cm^2^ fluence), whereas the total delivery of OS2966 1.68 ± 0.53 µg/cm^2^ ((Conditions: 300 pores over diffusion area, 35.3 J/cm^2^ fluence). However, it should be noted that there are technical and specification differences between the two laser devices. Another report by Cao *et al*. described the delivery of an anti-PD1 antibody against murine B16F10 melanoma after laser microporation with a CO_2_ laser^29^. However, there was no direct quantification of anti-PD1 and the endpoint was the measurement of tumour size: a significant reduction in tumour size was reported. Nevertheless, it has to be noted that murine skin has major structural differences with human skin: its epidermis has a mean thickness of 9.6 µm^30^, which is less than the thickness of human stratum corneum alone. The high follicle density is also a feature that is not representative of human skin^30^. Therefore, it is commonly accepted that mouse skin is not a representative surrogate for drug transport in human skin^30–33^.

In addition to confirming the feasibility of using fractional laser ablation to deliver OS2966, another key objective of the study was to determine the optimal poration conditions in order to move towards first-in-man experiments in the treatment of psoriasis (and other dermatological conditions). An optimal treatment would maximize selective delivery of the active into the skin, while minimizing transdermal permeation.

**Figure 7:**
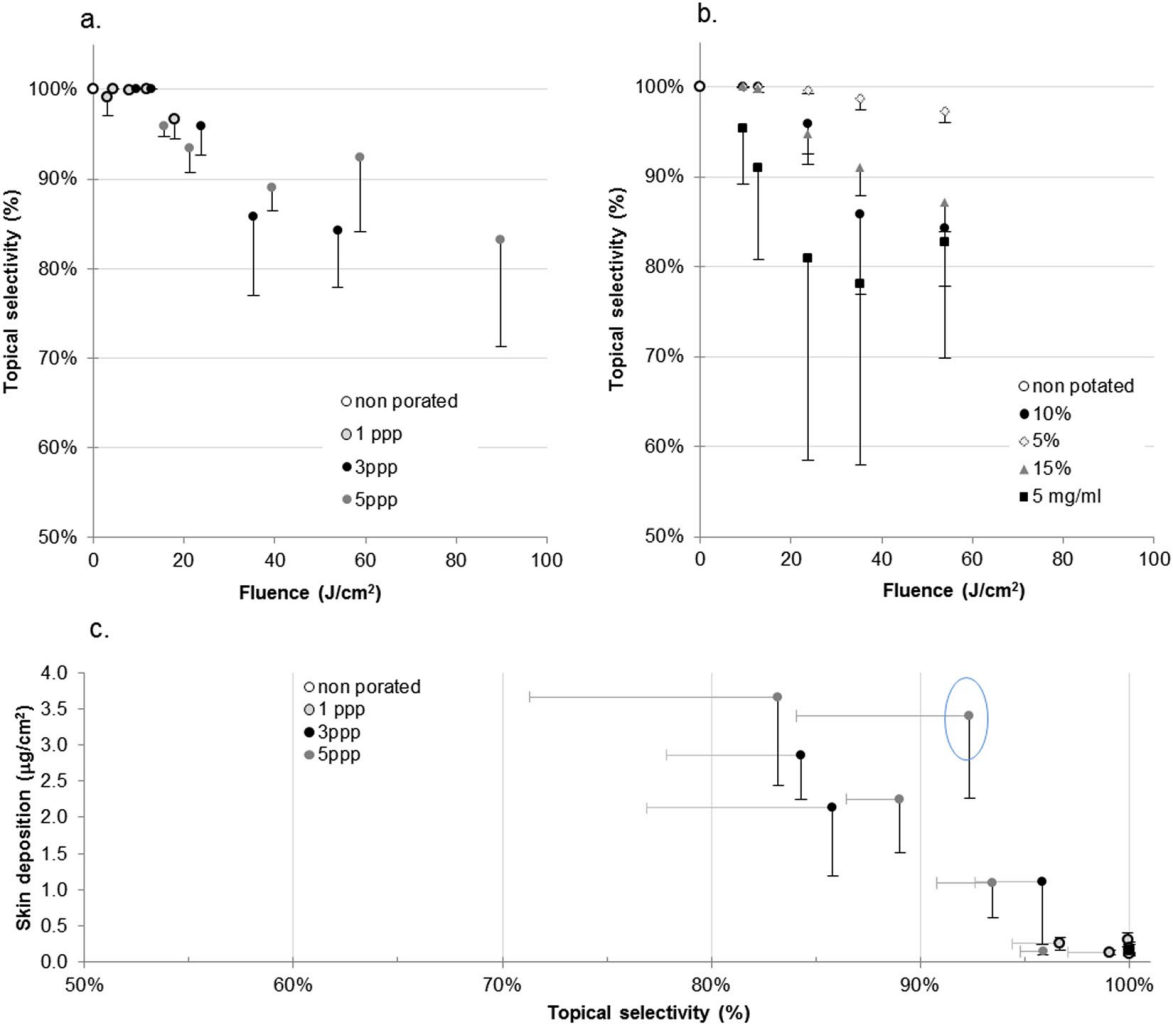
(**a**) Proportion of OS2966 delivered locally to skin relative to total delivery (topical selectivity) at a fixed FAA of 10%, (**b**) proportion of OS2966 delivered locally to skin relative to total delivery (topical selectivity) at 3 ppp with variation of the FAA and OS2966 concentration in the donor, (**c**) OS2966 skin deposition as a function of topical selectivity – a tool to select the optimal laser parameters for targeted cutaneous delivery (FAA of 10%, 1 mg/ml). ppp: pulse per pore.

Figure 7a shows the proportions of the OS2966 retained in the skin (i.e. delivered selectively into the skin) from a 1 mg/ml formulation at a FAA of 10% as a percentage of the total delivery, and it is worth noting that laser poration does allow selective targeting of the skin since even under the most aggressive conditions (89.9 J/cm^2^) as at least 83.2 ± 11.9% of the total active delivered, is delivered locally to the skin with upto 3.67 ± 1.22 µg/cm^2^ being recovered after formulation application for 12 h.

In the discussion below, the ability of the laser poration parameters as well as the formulation characteristics to target the skin will be referred to as the “topical selectivity” and expressed as the ratio of *skin deposition (µg/cm*^2^*)/skin deposition* + *skin permeation (µg/cm*^2^*)*.

Assuming that the volume of distribution for an antibody is approximately 4l as its distribution is limited to the blood compartment, as reported for infliximab^34^ and knowing that at fluences ≥35.3 J/cm^2^, OS2966 also permeates across skin and that cumulative permeation reaches 0.85 ± 0.40 μg/cm^2^ at the most aggressive condition, the blood concentration of OS2966 in this case would be 0.21 ng/ml after a 12 h application of a 1 mg/ml solution over a 1 cm^2^ pore array. Infliximab is a widely used monoclonal antibody for the treatment of severe psoriasis and can be used as a reference. The recommended by the FDA dose of infliximab is 5 mg/kg administered as an intravenous injection at 0, 2 and 6 weeks followed by a maintenance dose of 5 mg/kg every 8 weeks in case of chronic severe plaque psoriasis^35^. That means that for an average man of 70 kg, the single IV administered dose is 350 mg. Pharmacokinetic data in psoriatic patients shows that the first administration of a 5 mg/kg dose leads to infliximab blood levels reaching up to 100 µg/ml^34^. Consequently, even the most aggressive laser poration condition would lead to a 4.7 × 10^5^ -fold decrease in systemic exposure to the antibody whilst ensuring that microgramme amounts were present in the lesion.

When pore density and OS2966 content were varied (Fig. 7b**)**, the proportion of OS2966 delivered locally to skin remained high (above 78.0±20.1%); however, increasing the FAA to 15% neither significantly increased absolute delivery nor the topical selectivity of the treatment. Decreasing FAA to 5% yielded good topical selectivity, yet low skin deposition. Finally, increasing the formulation concentration increased absolute OS2966 delivery but at the cost of lower topical selectivity. The maximal skin permeation was obtained for the 5 mg/ml formulation (1.25 ± 0.46 µg/cm^2^) using the 3 ppp, 53.9 J/cm^2^ 10% FAA condition and is estimated to correspond to a plasma concentration of 0.31 ng/ml, which is still 5 orders of magnitude (10^5^) less than the maximal plasma levels in infliximab treated patients.

In order to select the optimal laser poration conditions it is interesting to plot the deposited OS2966 amounts against the topical selectivity (Fig. 7c**)**: the application of the 1 mg/ml formulation and poration at 10% FAA, 5 ppp, 58.8 J/cm^2^ appears to be the optimal treatment with more than 90% selectivity and 3.41 ± 1.14 µg/cm^2^ deposition.

OS2966 was successfully labelled with Alexa Fluor 488 dye in order to visualize the antibody penetration pathways. The diffusion of Alexa 488-OS2966 into the skin in the pore surroundings and beneath the pores was clearly higher than in intact skin. Increasing the fluence also resulted in stronger fluorescence in the pore regions, confirming the quantitative data. The optimal laser setting 10% FAA, 5 ppp, 58.8 J/cm^2^ yielded pores with a depth of150 μm, corresponding to the dermo-epithelial junction, meaning that OS2966 was able to reach its target and act on dermal T-cells about to cross the basal lamina. Finally, CLSM observation of hair follicles did not reveal significant involvement of the transfollicular pathway, meaning that diffusion through the experimentally-created pores was the preferential pathway for the mAb to enter the skin. In addition, the optimal laser condition of 5 ppp, 10%; 58.5 J/cm^2^, which was the second most aggressive condition, enabled the creation of micropores that nevertheless did not reach the dermis and its sensitive nerve endings.

It remains difficult to comment about an optimal laser condition as the therapeutic levels of OS2966 that have to be reached in skin are not yet known; Conrad *et al*.^23^. obtained clinically significant results in a xenotransplanted mouse *vivo* using a 1 mg mAb subcutaneous injection (twice weekly), therefore it needs to be confirmed that the amounts of OS2966 delivered to skin using laser poration are therapeutically relevant. Furthermore, it should be noted that the studies described here were conducted using aqueous solution and that an optimized formulation such as a gel will need to be developed in order to allow the topical application of OS2966 *in vivo* and better compliance of the patient.

Nevertheless, it has been clearly demonstrated that by tuning the laser settings, i.e. pore and pulse number and hence the applied energy, it is possible to control OS2966 delivery as well as to selectively target the skin. The antibody permeation yielded by laser treatment appears to be negligible since it was 10^5^ times lower than the plasma levels of infliximab after a single dose intravenous administration. In practice, the physio-pathological changes in psoriatic skin such as keratinization and scaling might necessitate further optimization of the laser treatment parameters.

## Conclusions

The results demonstrate the feasibility of delivering a 150,000 Da monoclonal antibody topically and selectively into the skin using the P.L.E.A.S.E.^®^ minimally-invasive laser assisted enhancement technique. These results are very promising for the treatment of psoriasis as a difficult-to-deliver, potent and selective biologic could be delivered to skin in a targeted way after topical application for 12 h, minimizing transdermal permeation and subsequent systemic exposure.

## Materials and Methods

### Materials

The monoclonal humanized antibody (OS2966), (5.87 mg/ml solution in PBS + Tween 20 0.1%) was supplied by OncoSynergy (South San Francisco, California, USA). The ELISA (enzyme-linked immunosorbent assay) Costar™ 96-well EIA/RIA plates were purchased from Fisher Scientific (Reinach, Switzerland). Tween 20 was purchased from Applichem Axon lab AG (Baden-Dättwil, Switzerland). HCl, NaCl, KCl, bovine serum albumin (BSA), Fab specific Anti-Human IgG, HRP (horseradish peroxidase) linked Fc specific Anti-Human IgG and 3,3′,5,5′-tetramethylbenzidine (TMB) liquid substrate were supplied by Sigma-Aldrich (Steinheim, Germany). Na_2_HPO_4_ and KH_2_PO_4_ were supplied by Acros Organics (Geel, Belgium). All other reagents were at least of reagent grade. All other solvents were HPLC grade (high performance liquid chromatography grade, HiPerSolv Chromatonorm; Darmstadt, Germany). Ultra-pure water (Millipore Milli-Q Gard 1 Purification Pack resistivity > 18MΩcm; Zug, Switzerland) was used to prepare all solutions.

### OS2966 quantification by enzyme-linked immunosorbent assay (ELISA)

OS2966 was quantified using enzyme-linked immunosorbent assay (ELISA). The coating Fab specific antibody (5 µg/ml in PBS; 100 µl) solution was introduced into each well of the Costar™ 96-well EIA/RIA plate and was covered with sealplate film and incubated at 37 °C for 60 min. The content of the plate was subsequently emptied and the plate was deposited upside down on absorbent paper for 1 min. The wells were washed once with 300 µl of wash buffer (PBS + Tween 20 0.1%). 250 µl of BSA 3% in wash buffer was used to block the non-specific binding sites. The plate was covered with sealplate film and incubated at 37 °C for 60 min. The wash step was subsequently repeated three times. Samples or standard were then introduced in the wells and incubated at room temperature (RT) for 120 min. The wash step was repeated three times. 100 µl of the solution of the HRP-linked antibody was introduced into each well and the plate was incubated at RT for 60 min. After a final wash step, TMB substrate was introduced into each well and the plate was incubated at RT in the dark for 5 min. The plate was read with the plate reader (BioTek Synergy Mx Multi-Mode microplate; BioTek, Luzern, Switzerland) at 620 nm. Finally, 100 µl of 1 N HCl solution was added to each well to stop the reaction and the plate was read again at 450 nm.

A standard curve was constructed using 0.1–1000 ng/ml concentrations of OS2966 in wash buffer. A five parameter logistic (5PL) function was used to perform the regression^36^. The fitting was performed using GraphPad Prism 6.03 software. R^2^ was superior to 0.99 for all of the regressions. The ELISA quantification method was validated regarding the specificity, selectivity, precision and accuracy (Supplementary Information Section 1). The limit of detection (LOD) and limit of quantification (LOQ) were determined to be 1 ng/ml and 3 ng/ml respectively.

### Skin source

Porcine ears were obtained from a local abattoir (CARRE; Rolle, Switzerland) within a few hours of sacrifice. Full thickness skin was harvested from the external side of the porcine ear. It was punched out to form discs with an area of 3 cm^2^, followed by trimming of any surface hair using clippers. The discs were stored at −20 °C for a maximum period of 30 days.

### Skin pre-treatment

Skin samples were equilibrated in 0.9% NaCl for 30 min prior to poration using the P.L.E.A.S.E.^®^ Professional device. After removing surface moisture, skin samples were placed at the focal length of the laser to create the micropores. Laser poration parameters, i.e., the fractional ablated area (FAA) and the fluence were fixed using the device software. The parameters were selected from the pre-programmed combinations set for dermatological applications to cover a range of fluences (Table 1**)**

**Table 1:** Different OS2966 content and laser poration parameters used. *FAA: Fractional ablated area; ppp: pulse per pore*.

OS2966 content	Stacking	FAA	Control	50µs/500 Hz	75µs/500 Hz	125µs/300 Hz	175µs/200 Hz	225µs/100 Hz
1 mg/ml	1 ppp	10%	0 J/cm^2^	3 J/cm^2^	4 J/cm^2^	7.9 J/cm^2^	11.7 J/cm^2^	17.8 J/cm^2^
1 mg/ml	3 ppp	10%		9 J/cm^2^	12 J/cm^2^	23.7 J/cm^2^	35.3 J/cm^2^	53.4 J/cm^2^
1 mg/ml	5 ppp	10%		15 J/cm^2^	20 J/cm^2^	39.5 J/cm^2^	58.5 J/cm^2^	89 J/cm^2^
1 mg/ml	3 ppp	5%		9 J/cm^2^	12 J/cm^2^	23.7 J/cm^2^	35.3 J/cm^2^	53.4 J/cm^2^
1 mg/ml	3 ppp	15%		9 J/cm^2^	12 J/cm^2^	23.7 J/cm^2^	35.3 J/cm^2^	53.4 J/cm^2^
5 mg/ml	3ppp	10%	0 J/cm^2^	9 J/cm^2^	12 J/cm^2^	23.7 J/cm^2^	35.3 J/cm^2^	53.4 J/cm^2^

### Delivery experiments: experimental set-up

Skin samples with or without laser pre-treatment were clamped in Franz-diffusion cells (diffusion area 2.0 cm^2^) and the receiver compartment was filled with 10 ml of PBS pH 7.4 + Tween 20 0.1%. The diffusion cells were placed in a water bath at 33 °C and the receiver solution was stirred at 250 rpm. 500 µl of OS2966 solution (1 mg/ml or 5 mg/ml in PBS) were placed in the donor compartment and contact of the formulation with the skin surface was checked. Each group consisted of 5 replicates, a cell with PBS as donor solution was used as the negative control. The cells were finally covered with Parafilm™; the duration of the permeation experiments was 12 h. At the end of the experiment, 1 ml was collected from the receiver compartment to quantify OS2966 skin permeation. Then, the diffusion cells were dismantled, the excess formulation removed from the skin surface with a tissue and the skin washed twice with a water impregnated cotton bud. Finally, the skin surface was dried with a soft tissue. OS2966 deposited in the skin was extracted by cutting the tissue into small pieces and soaking them in 10 ml of PBS pH 7.4 + Tween 20 0.1% for 4 hours under constant agitation at room temperature. The samples were centrifuged and appropriately diluted before undergoing ELISA analysis.

### Data analysis

Skin delivery data were expressed as mean ± SD. Outliers were determined using the Dixon’s Q test (α = 0.05) and discarded. Results were evaluated statistically using SigmaPlot v.11.0. Groups were compared using either analysis of variance (ANOVA) or analysis of means by Student’s t-test. Student Newman Keuls test or Bonferroni T-test were used when necessary as post-hoc procedures. The level of significance was fixed at α = 0.05. (the detailed statistics are presented in Supplementary Information Section 3).

### Fluorescent labelling of OS2966

The OS2966 was labelled using Alexa Fluor^®^ 488 Protein Labeling Kit (Molecular probes^®^, life technologies A-10235).

The labelled OS2966 was purified on Bio-Rad BioGel P-30 size exclusion purification resin provided in the kit. Fractions of ~200 μl were collected in Eppendorf tubes. At the end of purification, all tubes were observed under a UV lamp at 366 nm to visualize the tubes containing fluorescent compounds. The labelled antibody (Alexa 488-OS2966) was well separated from the residual free dye and collected in fractions 8–12 (Supplementary Information Section 2). The calculated concentration of the Alexa 488-OS2966 in the pooled 8 to 12 fractions was 1.4 mg/ml. This concentration was subsequently adjusted to 1 mg/ml for skin delivery experiments.

All factions were tested by HPLC-Fluo for the presence of the free dye. Free dye was quantified using an HPLC system (formerly Dionex AG, now Thermo Fisher Scientific AG; Reinach Switzerland) composed of a P680A LPG-4 pump, an ASI-100 autosampler, a thermostatted column compartment TCC-100, a UV170U detector and a RF 2000 fluorescence detector. Data collection and integration were achieved using Chromeleon® (version 6.8) software.

A Lichrospher RP-C8, 5 μm column (4,6 × 125 mm) thermostatted at 40 °C was used for the separation. The mobile phase consisted of a 70:30 mixture of methanol:water. Analyses were performed using an injection volume of 25 μl and a flow rate of 0.6 ml/min. The excitation wavelength was set at 494 nm and the emission wavelength at 519 nm. A peak for Alexa fluor 488 was obtained at 2.518 min. The Alexa 488-OS2966 in the pooled 8 to 12 fractions was found to be 99.4% pure (m/m) (Supplementary Information Section 2).

### Confocal laser scanning microscopy visualization (CLSM)

The delivery experiments for the confocal microscopy study were performed using the same conditions as for the “conventional” quantitative delivery experiments. 1 mg/ml solution of Alexa 488-OS2966 in PBS was applied to the donor compartment. Only the stacking was varied from 1 ppp to 5 ppp leading to fluences ranging from 11.7 J/cm^2^ to 58.5 J/cm^2^.

After the permeation experiment, cells were dismantled and skin samples were deposited on cotton swabs impregnated with a Hoechst 33258 solution (100 µg/ml). Hoechst 33258 was allowed to diffuse into the skin from the dermal side for 30 min in order to stain dermal and epidermal tissue.

Finally, the skin was cut in lateral slices (approx. 1 mm thick) with a scalpel and mounted on a glass slide in two ways: either *stratum corneum* (xy-plane) facing the microscope objective (LSM 710, Zeiss, Germany), or the vertical cross-section (xz-plane) slice facing the microscope objective. (Supplementary Information Section 3).

The confocal images were obtained using the settings mentioned in Table 2 with an Achroplan 20x air or a Fluar 5x objective and analyzed using Image J software. Each image was the average of 4 repeated scans.

**Table 2:** CLSM settings for both dyes.

	Alexa fluor 488	Hoechst 33258
Excitation Laser	488 nm	405 nm
Emission filter	510–530 nm	431–460 nm
Laser power	20	60
Pinhole	100	300
Master gain	500	600

The morphology of the pores created with the optimal laser treatment was confirmed by optical microscopy after a standard Hematoxylin and eosin staining procedure.

## Supplementary information

Supplemenatary Information
