# Supplementary material for: Fractional laser ablation for the targeted cutaneous delivery of an anti-CD29 monoclonal antibody – OS2966
Source: Sci Rep. 2019 Jan 31;9:1030. doi: 10.1038/s41598-018-36966-0 (PMC6355906; doi:10.1038/s41598-018-36966-0)
Supplement: Supplementary file 1 — Supplemenatary Information [file 41598_2018_36966_MOESM1_ESM.docx]

Fractional laser ablation for the targeted cutaneous delivery of an anti-CD29 monoclonal antibody – OS2966

Supplementary Information

*Maria Lapteva^1^ Sergio del Río-Sancho^1^, Eric Wu^2^, W. Shawn Carbonell^2^, Christof Böhler^3^, Yogeshvar N. Kalia^1^*

^1^School of Pharmaceutical Sciences, University of Geneva & University of Lausanne, Geneva, Switzerland

^2^ OncoSynergy, Inc., 329 Oyster Point Blvd, South San Francisco, USA

^3^ Pantec Biosolutions, Ruggell, Lichtenstein.

**Corresponding author**:

Prof. Yogeshvar N. Kalia

School of Pharmaceutical Sciences

University of Geneva

CMU, 1 rue Michel Servet

CH-1211 Geneva 4, Switzerland

Tel.: +41 22 379 3355

Fax: +41 22 379 3360

Email: yogi.kalia@unige.ch

# ELISA method validation

## Data analysis

A standard curve was constructed using 0.1 – 1000 ng/ml concentrations of OS2966 in wash buffer. **Figure S1** presents a typical calibration curve.

**Figure S1.** Representative OS2966 calibration curve and prediction yielded by the 5PL regression.

The five parameter logistic (5PL) function was used to perform the regression[^1^](#_ENREF_1). The function is described by **Equation S1**.

| $Abs=d+ \frac{(a-d)}{{{(1+(\frac{[OS2966]}{c})}^{b})}^{e}}$ | *Equation (S1)* |
| --- | --- |

With

| a | the minimum asymptote |
| --- | --- |
| b | the slope at inflection point |
| c | the concentration at inflection point |
| d | the maximum asymptote |
| e | an asymmetry parameter |

The fitting was performed using **GraphPad Prism 6.03** software. R^2^ was superior to 0.99 for all of the regressions. The OS2966 content in skin extraction and skin permeation samples was calculated using Equation S2:

| $[OS2966]= {{c \times(\left( \frac{a-d}{Abs-d} \right)}^{\frac{1}{e}}-1)}^{\frac{1}{b}}$ | *Equation (S2)* |
| --- | --- |

## Specificity, matrix effect, LOD and LOQ.

No interference from skin was observed when performing the calibration curves in different media (**Figure S2**), meaning that no matrix effect was occurring.

**Figure S2.** OS2966 calibration curves in different media.(mean ± SD, n=3)

The limit of detection (lowest standard to be statistically different from the blank signal) contained 1 ng/ml of OS2966. Therefore, the limit of detection (LOD) and limit of quantification (LOQ) were determined to be 1 ng/ml and 3 ng/ml, respectively.

## Precision and accuracy

The intra-day / intra-plate variability was not measured as every plate had its own calibration curve. The inter-day / inter-plate precision and accuracy is presented in **Table S1**.

**Table S1.** Precision and accuracy of the analytical method.

|  | Inter-day (3 days) | | |
| --- | --- | --- | --- |
| [OS2966]theo (ng/ml) | [OS2966]meas (mean ± SD in ng/ml) | RSD (%) | Recovery (%) |
| 1 | 1.1 ± 0.1 | 5.9 | 105.5 |
| 20 | 20.0 ± 0.9 | 4.7 | 99.8 |
| 100 | 97.1 ± 3.6 | 3.7 | 97.1 |

The results obtained were within the acceptance limits of analytical method validation guidelines [^2^](#_ENREF_2)^,^[^3^](#_ENREF_3); therefore, the ELISA method was considered to be precise and accurate.

# Fluorescent labelling of OS2966

## Selection of fractions

At the end of purification, all tubes were observed under a UV lamp at 366 nm to visualize the tubes containing fluorescent compounds.


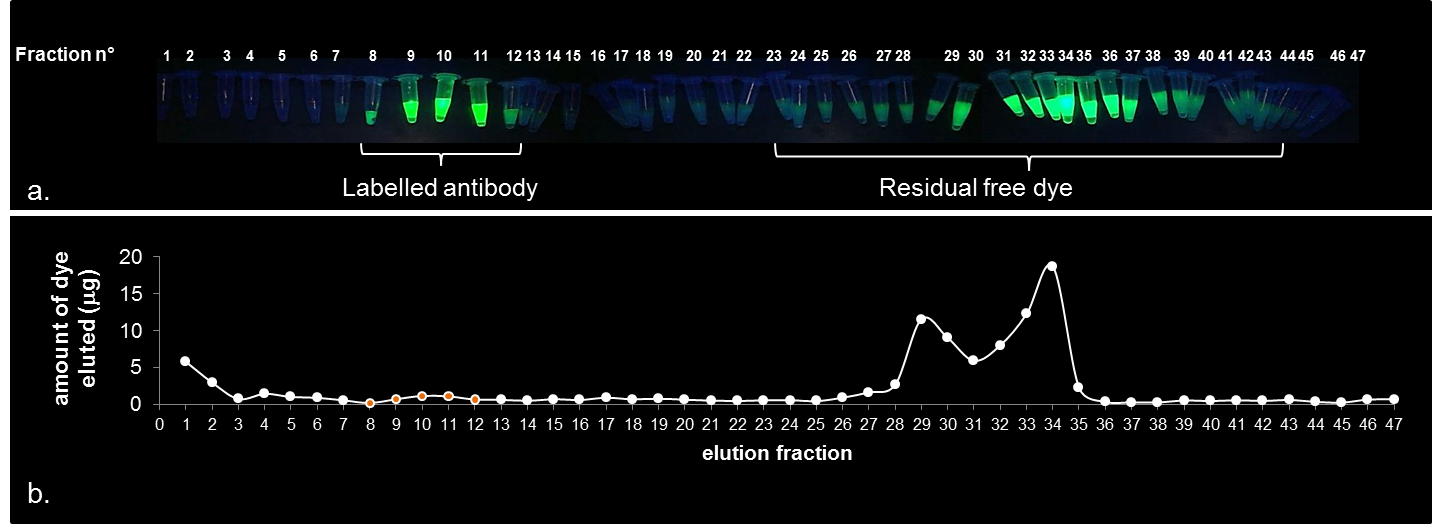


**Figure S3.** a) Fluorescence after excitation at 366 nm in fractions collected after purification step. b) Quantification of Alexa 488 free dye in all fractions by HPLC – Fluo.

As evidenced by the observation of the fractions under the under UV lamp (**Figure S3a**), the labelled antibody (Alexa 488-OS2966) was mainly in fractions 8-12, well separated from the residual free dye in fractions 17-45. Thus, fractions 8-12, containing Alexa 488-OS2966, were pooled.

## Estimation of Alexa 488-OS2966 concentration and degree of labelling

The Alexa 488-OS2966 was diluted 1:10 and its absorbance was measured at 280 and 494 nm using a 1 cm path-length quartz cuvette. The protein content and the degree of labelling (DoL) were calculated using the following equations:

| $protein concentration (M) = \frac{(A_{280}{-(A}_{494}\times0.11)) \times dilution factor}{203000}$ | *Equation (S3)* |
| --- | --- |
| $DoL (moles dye per moles of protein)= \frac{A_{494}\times dilution factor}{71000\times protein concentration (M)}$ | *Equation (S4)* |

where

| A280 | is the absorbance of diluted Alexa 488-OS2966 at 280 nm |
| --- | --- |
| A494 | is the absorbance of diluted Alexa 488-OS2966 at 494 nm |
| 203000 | is the molar extinction coefficient of IgG at 280 nm |
| 0.11 | is a correction factor to account for absorption of the dye at 280 nm. |
| 71000 | is the molar extinction coefficient of Alexa dye at 494 |

As a result, the Alexa 488-OS2966 content in pooled fractions 8 to 12 was **1.4 mg/ml** (considering the MW of a conventional IgG = 150000 Da) and the DoL was **4.8 mole of dye per mole of OS2966.**

## Quantification of free dye

All fractions were tested by HPLC-Fluo for the presence of the free dye. Free dye was quantified using a Lichrospher RP-C8, 5 μm column (4,6 × 125 mm) thermostatted at 40°C. The mobile phase consisted of a 70:30 mixture of methanol:water. Analyses were performed using an injection volume of 25 μl and a flow rate of 0.6 ml/min. The excitation wavelength was set at 494 nm and the emission wavelength at 519 nm. A peak for Alexa Fluor 488 was obtained at 2.518 min. Figure S3b shows the distribution of the free dye in the different elution fractions.

The free dye clearly elutes in fractions 26-36 and is well separated from the labelled antibody. The amount of free dye found in fractions 8-12 was estimated at 3.8 μg, meaning that the protein was 99.4% pure (m/m)

## Skin sample preparation


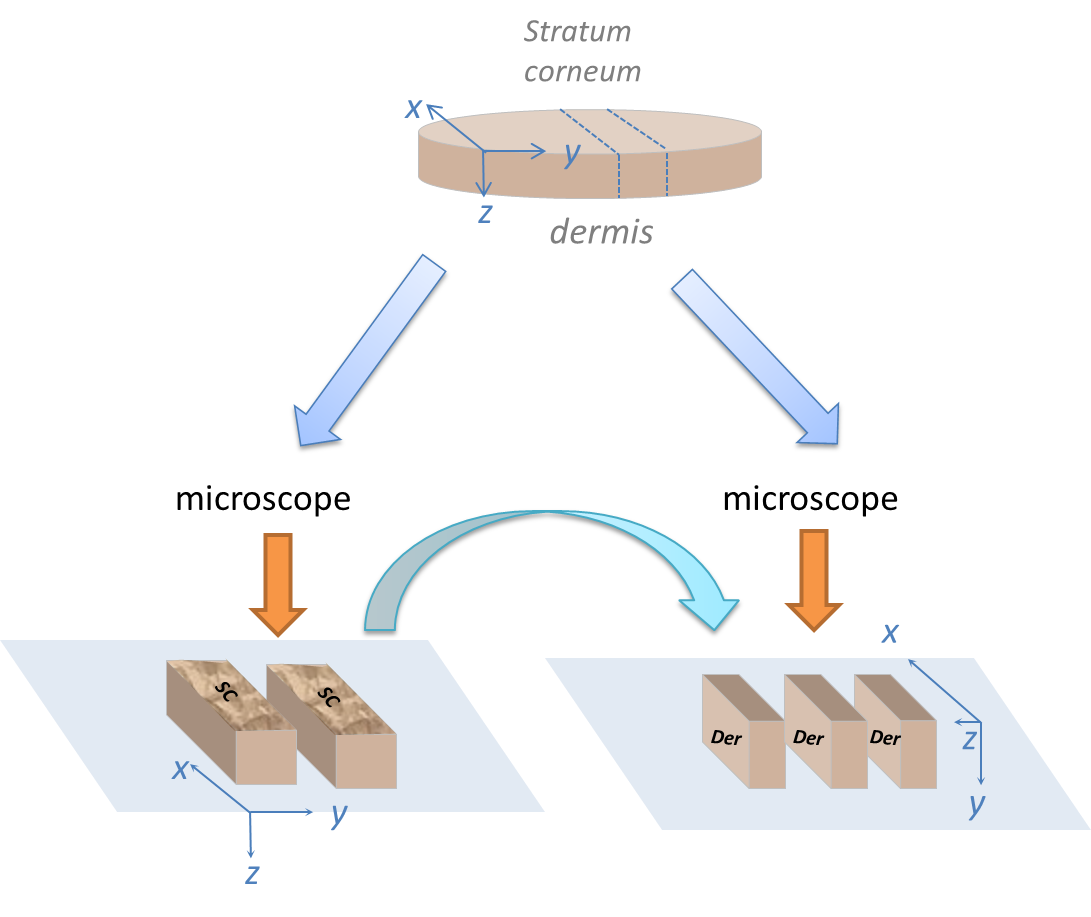


**Figure S4**. Skin sample preparation prior to microscopic observation.

# Statistical analysis

This section summarizes the statistical comparison of all data.

## Comparison of laser poration parameters at fixed donor concentration and FAA

**Table S2.** Significance in difference for skin deposition data (SNK multiple comparison, pairwise)

| Fluence (J/cm^2^) | **0.0** | **3.1** | **4.3** | **7.9** | **11.8** | **18.0** | **9.4** | **12.8** | **23.7** | **35.3** | **53.9** | **15.6** | **21.3** | **39.5** | **58.8** | **89.9** |
| --- | --- | --- | --- | --- | --- | --- | --- | --- | --- | --- | --- | --- | --- | --- | --- | --- |
| **0.0** |  | **0.9600** | **1.0000** | **1.0000** | **1.0000** | **1.0000** | **0.9980** | **1.0000** | **0.8560** | **<0.001** | **<0.001** | **1.0000** | **0.3130** | **<0.001** | **<0.001** | **<0.001** |
| **3.1** |  |  | 1.0000 | 1.0000 | 1.0000 | 1.0000 | 0.9880 | 1.0000 | 0.7980 | <0.001 | <0.001 | 1.0000 | 0.2300 | <0.001 | <0.001 | <0.001 |
| **4.3** |  |  |  | 0.9950 | 0.9960 | 0.9960 | 0.9960 | 0.9880 | 0.6560 | <0.001 | <0.001 | 0.9480 | 0.1690 | <0.001 | <0.001 | <0.001 |
| **7.9** |  |  |  |  | 0.9520 | 0.8850 | 0.9990 | 0.9850 | 0.2540 | <0.001 | <0.001 | 0.9980 | 0.0910 | <0.001 | <0.001 | <0.001 |
| **11.8** |  |  |  |  |  | 0.8930 | 1.0000 | 0.9470 | 0.4850 | <0.001 | <0.001 | 0.9990 | 0.1190 | <0.001 | <0.001 | <0.001 |
| **18.0** |  |  |  |  |  |  | 1.0000 | 0.9800 | 0.4400 | <0.001 | <0.001 | 0.9990 | 0.1460 | <0.001 | <0.001 | <0.001 |
| **9.4** |  |  |  |  |  |  |  | 1.0000 | 0.7530 | <0.001 | <0.001 | 0.9860 | 0.2050 | <0.001 | <0.001 | <0.001 |
| **12.8** |  |  |  |  |  |  |  |  | 0.6160 | <0.001 | <0.001 | 0.9970 | 0.1850 | <0.001 | <0.001 | <0.001 |
| **23.7** |  |  |  |  |  |  |  |  |  | 0.0020 | <0.001 | 0.6980 | 0.3900 | 0.0070 | <0.001 | <0.001 |
| **35.3** |  |  |  |  |  |  |  |  |  |  | 0.1220 | <0.001 | 0.0020 | 0.7430 | 0.0050 | <0.001 |
| **53.9** |  |  |  |  |  |  |  |  |  |  |  | <0.001 | <0.001 | 0.1010 | 0.1430 | 0.0810 |
| **15.6** |  |  |  |  |  |  |  |  |  |  |  |  | 0.1800 | <0.001 | <0.001 | <0.001 |
| **21.3** |  |  |  |  |  |  |  |  |  |  |  |  |  | 0.0070 | <0.001 | <0.001 |
| **39.5** |  |  |  |  |  |  |  |  |  |  |  |  |  |  | 0.0070 | 0.0020 |
| **58.8** |  |  |  |  |  |  |  |  |  |  |  |  |  |  |  | 0.4840 |
| **89.9** |  |  |  |  |  |  |  |  |  |  |  |  |  |  |  |  |

**Table S3.** Skin deposition data: Multiple Comparisons versus Control Group (Bonferroni t-test)

| Comparison | P |
| --- | --- |
| 0.0 vs. 89.9 | <0.001 |
| 0.0 vs. 58.8 | <0.001 |
| 0.0 vs. 53.9 | <0.001 |
| 0.0 vs. 39.5 | <0.001 |
| 0.0 vs. 35.3 | <0.001 |
| 0.0 vs. 21.3 | 0.215 |
| 0.0 vs. 23.7 | 1 |
| 0.0 vs. 7.9 | 1 |
| 0.0 vs. 18.0 | 1 |
| 0.0 vs. 11.8 | 1 |
| 0.0 vs. 12.8 | 1 |
| 0.0 vs. 4.3 | 1 |
| 0.0 vs. 15.6 | 1 |
| 0.0 vs. 9.4 | 1 |
| 0.0 vs. 3.1 | 1 |

**Table S4.** Significance in difference for skin permeation data (SNK multiple comparison, pairwise)

| Fluence (J/cm^2^) | **0.0** | **3.1** | **4.3** | **7.9** | **11.8** | **18.0** | **9.4** | **12.8** | **23.7** | **35.3** | **53.9** | **15.6** | **21.3** | **39.5** | **58.8** | **89.9** |
| --- | --- | --- | --- | --- | --- | --- | --- | --- | --- | --- | --- | --- | --- | --- | --- | --- |
| **0.0** |  | **1.0000** | **1.0000** | **1.0000** | **1.0000** | **1.0000** | **1.0000** | **1.0000** | **0.9600** | **<0.001** | **<0.001** | **1.0000** | **0.9600** | **0.0180** | **0.0070** | **<0.001** |
| **3.1** |  |  | 1.0000 | 0.9920 | 1.0000 | 0.9940 | 1.0000 | 1.0000 | 0.8270 | <0.001 | <0.001 | 0.9490 | 0.8270 | 0.0090 | 0.0040 | <0.001 |
| **4.3** |  |  |  | 1.0000 | 1.0000 | 1.0000 | 1.0000 | 1.0000 | 0.9860 | <0.001 | <0.001 | 1.0000 | 0.9860 | 0.0260 | 0.0100 | <0.001 |
| **7.9** |  |  |  |  | 1.0000 | 0.9990 | 1.0000 | 1.0000 | 0.8920 | <0.001 | <0.001 | 0.9970 | 0.8920 | 0.0110 | 0.0050 | <0.001 |
| **11.8** |  |  |  |  |  | 1.0000 | 1.0000 | 1.0000 | 0.9760 | <0.001 | <0.001 | 1.0000 | 0.9760 | 0.0220 | 0.0080 | <0.001 |
| **18.0** |  |  |  |  |  |  | 1.0000 | 0.1690 | 0.8030 | <0.001 | <0.001 | 0.9630 | 0.6490 | 0.0100 | 0.0070 | <0.001 |
| **9.4** |  |  |  |  |  |  |  | 1.0000 | 0.9340 | <0.001 | <0.001 | 1.0000 | 0.9340 | 0.0100 | 0.0050 | <0.001 |
| **12.8** |  |  |  |  |  |  |  |  | 0.9860 | <0.001 | <0.001 | 1.0000 | 0.9860 | 0.0260 | 0.0100 | <0.001 |
| **23.7** |  |  |  |  |  |  |  |  |  | <0.001 | <0.001 | 0.9480 | 0.5340 | 0.0110 | 0.0060 | <0.001 |
| **35.3** |  |  |  |  |  |  |  |  |  |  | 0.4170 | <0.001 | <0.001 | 0.1330 | 0.2000 | <0.001 |
| **53.9** |  |  |  |  |  |  |  |  |  |  |  | <0.001 | <0.001 | 0.0240 | 0.5600 | <0.001 |
| **15.6** |  |  |  |  |  |  |  |  |  |  |  |  | 0.7540 | 0.0070 | 0.0030 | <0.001 |
| **21.3** |  |  |  |  |  |  |  |  |  |  |  |  |  | 0.0140 | 0.0120 | <0.001 |
| **39.5** |  |  |  |  |  |  |  |  |  |  |  |  |  |  | 0.2000 | <0.001 |
| **58.8** |  |  |  |  |  |  |  |  |  |  |  |  |  |  |  | <0.001 |
| **89.9** |  |  |  |  |  |  |  |  |  |  |  |  |  |  |  |  |

**Table S5.** Skin permeation data: Multiple Comparisons versus Control Group (Bonferroni t-test)

| Comparison | P |
| --- | --- |
| 0.0 vs. 89.9 | <0.001 |
| 0.0 vs. 58.8 | <0.001 |
| 0.0 vs. 53.9 | <0.001 |
| 0.0 vs. 39.5 | 0.003 |
| 0.0 vs. 35.3 | 0.01 |
| 0.0 vs. 21.3 | 1 |
| 0.0 vs. 23.7 | 1 |
| 0.0 vs. 7.9 | 1 |
| 0.0 vs. 18.0 | 1 |
| 0.0 vs. 11.8 | 1 |
| 0.0 vs. 12.8 | 1 |
| 0.0 vs. 4.3 | 1 |
| 0.0 vs. 15.6 | 1 |
| 0.0 vs. 9.4 | 1 |
| 0.0 vs. 3.1 | 1 |

## Comparison of different fractional ablated area using 3ppp and 1 mg/ml donor

**Table S6.** Significance in difference for skin deposition data

| Compared groups (FAA; Fluence (J/cm^2^)) | | | ANOVA *p value* | SNK result | | |
| --- | --- | --- | --- | --- | --- | --- |
|  |  |  |  | groups | *p value* | significance |
| 5% ; 9.4 | 10 % ; 9.4 | 15% ; 9.4 | 0.542 | -- | | |
| 5% ; 12.8 | 10% ; 12.8 | 15% ; 12.8 | 0.887 | -- | | |
| 5% ; 23.7 | 10% ; 23.7 | 15% ; 23.7 | 0.046 | 10% vs. 5% | 0.047 | Yes |
|  |  |  |  | 10% vs. 15% | 0.056 | No |
|  |  |  |  | 15% vs. 5% | 0.538 | No |
| 5% ; 35.3 | 10% 35.3 | 15% 35.3 | <0.001 | 10% vs. 5% | <0.001 | Yes |
|  |  |  |  | 10% vs. 15% | 0.128 | No |
|  |  |  |  | 15% vs. 5% | 0.005 | Yes |
| 5% ; 53.9 | 10% 53.9 | 15% 53.9 | <0.001 | 10% vs. 5% | <0.001 | Yes |
|  |  |  |  | 10% vs. 15% | 0.79 | No |
|  |  |  |  | 15% vs. 5% | <0.001 | Yes |

**Table S7.** Significance in difference for skin permeation data

| Compared groups (FAA; Fluence (J/cm^2^)) | | | ANOVA *p value* | SNK result | | |
| --- | --- | --- | --- | --- | --- | --- |
|  |  |  |  | groups | *p value* | significance |
| 5% ; 9.4 | 10 % ; 9.4 | 15% ; 9.4 | 0.397 | -- | | |
| 5% ; 12.8 | 10% ; 12.8 | 15% ; 12.8 | 0.281 | -- | | |
| 5% ; 23.7 | 10% ; 23.7 | 15% ; 23.7 | 0.018 | 10% vs. 5% | 0.029 | Yes |
|  |  |  |  | 10% vs. 15% | 0.896 | No |
|  |  |  |  | 15% vs. 5% | 0.015 | Yes |
| 5% ; 35.3 | 10% 35.3 | 15% 35.3 | 0.008 | 10% vs. 5% | 0.006 | Yes |
|  |  |  |  | 10% vs. 15% | 0.057 | No |
|  |  |  |  | 15% vs. 5% | 0.112 | No |
| 5% ; 53.9 | 10% 53.9 | 15% 53.9 | <0.001 | 10% vs. 5% | 0.001 | Yes |
|  |  |  |  | 10% vs. 15% | 0.417 | No |
|  |  |  |  | 15% vs. 5% | 0.002 | Yes |

## Comparison of different donor concentration using 3ppp, 10% FAA

**Table S8.** Significance when comparing different donor solutions

|  | **T-test p-values when comparing 1 mg/ml to 5 mg/ml donnor** | | | | | |
| --- | --- | --- | --- | --- | --- | --- |
| Fluence (J/cm^2^) | **0** | **9.4** | **12.8** | **23.7** | **35.3** | **53.9** |
| Deposition | 0.281 | 0.046 | 0.001 | 0.070 | 0.094 | 0.098 |
| Permeation | 0.122 | 0.322 | 0.131 | 0.119 | 0.278 | 0.044 |

# References

1 Gottschalk, P. G. & Dunn, J. R. The five-parameter logistic: a characterization and comparison with the four-parameter logistic. *Anal Biochem.* **343**, 54-65 (2005).

2 *Guidance for Industry: Bioanalytical Method Validation*, (2001) Available at: <http://www.fda.gov/downloads/Drugs/.../Guidances/ucm070107.pdf>. (Accessed: 27.05.13)

3 *Validation of Analytical Procedures: Text and Methodology Topic Q 2 (R1)*, (2005) Available at: <http://www.ich.org/fileadmin/Public_Web_Site/ICH_Products/Guidelines/Quality/Q2_R1/Step4/Q2_R1__Guideline.pdf>. (Accessed: 27.05.13)
